# Agreement of objectively measured physical activity and sedentary time in preschool children

**DOI:** 10.1016/j.pmedr.2015.07.009

**Published:** 2015-07-21

**Authors:** Eivind Aadland, Kjersti Johannessen

**Affiliations:** Faculty of Teacher Education and Sport, Sogn og Fjordane University College, Box 133, 6851 Sogndal, Norway

**Keywords:** Test–retest, Bland–Altman plot, Measurement, Accelerometry

## Abstract

**Objective:**

To determine the intra-individual agreement for objectively measured physical activity (PA) and sedentary behavior (SED) over two subsequent weeks in preschool children.

**Method:**

Ninety-one children aged 3 to 5 years (49% boys) from three preschools in Sogn og Fjordane, Norway, provided 14 consecutive days of accelerometer data (Actigraph GT3X +) during the autumn of 2014. Week-by-week reliability was assessed using intraclass correlation (ICC), Bland–Altman plots and 95% limits of agreement for different wear time criteria (≥ 6, 8 and 10 h/day and ≥ 3 and 5 days/week).

**Results:**

The week-by-week ICC was ≥ 0.75 for all variables across all wear criteria applied, except for absolute sedentary time (ICC 0.61–0.81). Using a ≥ 8 h/day and ≥ 3 days/week criterion (n = 78), limits of agreement were ± 209.5 cpm for overall PA, ± 68.6 min/day for SED, ± 43.8 min/day for light PA, ± 20.2 min/day for moderate-to-vigorous PA, and ± 55.9 min/day for light-to-vigorous PA, equaling 1.0–1.6 standard deviation units.

**Conclusion:**

Considerable week-by-week variability was found for all variables. Researchers need to be aware of substantial intra-individual variability in accelerometer-measurements and take necessary actions according to the hypothesis under study, as noise in any measurement will preclude researchers' ability to arrive at valid conclusions in epidemiology.

## Highlights

• Measurement error is a challenge in epidemiology.
• Accelerometers are highly used to assess physical activity level.
• Agreement for such measurements has not been determined.
• Physical activity and sedentary time varies substantially over time in preschoolers.
• Good methodological considerations are essential to arrive at valid conclusions.

## Introduction

Objective assessment of movement is the cornerstone of most ongoing epidemiological studies investigating health benefits of physical activity (PA) and sedentary behavior (SED). Yet, measurement error may preclude researchers from arriving at valid conclusions and possibly misinform the society regarding targets for public health initiatives (Hutcheon et al., 2010). Given the inherent variation in behavior over time, an important aspect of accelerometer measurements is how many days of measurement that are needed to obtain reliable estimates of habitual activity level.

Although findings vary somewhat between studies in both adults (Coleman and Epstein, 1998, Gretebeck and Montoye, 1992, Hart et al., 2011, Jerome et al., 2009, Matthews et al., 2002, Trost et al., 2005) and children (Addy et al., 2014, Basterfield et al., 2011, Hinkley et al., 2012, Hislop et al., 2014, Janz et al., 1995, Kang et al., 2009, Murray et al., 2004, Ojiambo et al., 2011, Penpraze et al., 2006, Rich et al., 2013, Treuth et al., 2003, Trost et al., 2000), most evidence suggest that a reliability (i.e., intraclass correlation (ICC)) of ~ 0.70–0.80 are achieved with 3–7 days of monitoring by estimation of the reliability and the number of days needed based on the Spearman Brown prophecy formula when measurements are conducted over a single 7-day period. However, such study designs have been criticized for possibly leading to optimistic results and should be interpreted with caution (Baranowski et al., 2008, Matthews et al., 2012, Wickel and Welk, 2010). First, the results are in principle only generalizable to the included days, as inclusion of additional days, weeks or seasons will add variability. Some few studies have determined the reliability for several periods of measurement over the course of a year, of which all have shown considerable intra-individual variation (Levin et al., 1999, Mattocks et al., 2007, Wickel and Welk, 2010), leaving reliability estimates for ~ 0.50 for one week monitoring in children. Second, the assumption of compound symmetry (i.e., similar variances and co-variances across days of measurement) might not be fulfilled. Additionally, ICC is the variance partitioning of subjects to the total variance, thus ICC is a relative and context-specific estimate that depends on the heterogeneity of the sample (Bland and Altman, 1986, Hopkins, 2000, Weir, 2005).

No studies have determined the intra-individual week-by-week agreement of accelerometer outcomes using absolute measures of reliability, i.e., standard error of the measurement (SEM) or limits of agreement (LoA). Such measures provide researchers a direct quantification of how much outcomes should be expected to vary over time and is independent of the variability of observations (Bland and Altman, 1986, Hopkins, 2000, Weir, 2005).

Consistent with studies in other age groups, it is estimated that ~ 3–7 days of accelerometer monitoring are needed to reliably determine PA in preschool children (Addy et al., 2014, Hinkley et al., 2012, Hislop et al., 2014, Penpraze et al., 2006). As preschool children is an understudied population in PA epidemiology (Pate et al., 2013), the quantification of measurement error for determination of PA and SED in this age-group is important for methodological considerations concerning the measurement of habitual activity level, which is fundamental to promote high-quality research and significantly advance knowledge in this field.

The aim of the present study was to determine the intra-individual agreement of PA and SED for two subsequent weeks of measurement in preschool children. Based on previous studies, we hypothesized great variability across weeks for all accelerometer outcomes.

## Methods

### Subjects

Ninety-four children aged 3 to 5 years from three different preschools in the county of Sogn og Fjordane, Norway were recruited for a two-week objective measurement of PA level during the autumn 2014. Written informed consent was obtained from the children's parents/guardians prior to the data collection. The study was approved by the Norwegian Social Science Data Services.

### Procedures

Physical activity was measured using the Actigraph GT3X + accelerometer (firmware 2.2.1) (Pensacola, FL, USA) (John and Freedson, 2012). Children were instructed to wear the accelerometer at all times over two consecutive weeks, except during water activities (swimming, showering) or while sleeping. Parents/guardians and preschool personnel were encouraged to be vigilant concerning the use of the accelerometers every day for the 14 day period. Units were initialized at a sampling rate of 30 Hz. Files were analyzed at 10 second epochs using Kinesoft© v. 3.3.75 software (Kinesoft), using different criteria for valid wear time (≥ 6; ≥ 8; ≥ 10 h/day). In all analyses, consecutive periods of ≥ 20 min of zero counts were defined as non-wear time (Cain et al., 2013, Esliger et al., 2005). Results are reported for overall PA level (cpm), as well as SED (< 100 cpm), light PA (LPA) (100–2295 cpm), moderate-to-vigorous PA (MVPA) (≥ 2296 cpm) and light-to-vigorous PA (LVPA) (non-SED PA) (≥ 100 cpm) obtained from the vertical axis (axis 1) (Evenson et al., 2008, Janssen et al., 2013, Trost et al., 2011). Intensity-specific PA and SED were reported as min/day and as percentage values of valid wear time.

### Statistical analyses

Subject characteristics were reported as frequencies, means and standard deviations (SD).

The single-day reliability and number of days needed to obtain the desired reliability were determined for wear times of ≥ 6, ≥ 8 and ≥ 10 h/day. Reliability for single days of measurement was assessed using variance partitioning obtained through a one-way random effect model (between subject variance / (between subject variance + residual variance)) (McGraw and Wong, 1996). Number of days needed to obtain a reliability of 0.80 was estimated using the Spearman Brown prophecy formula/ICC for average measurements (McGraw and Wong, 1996, Trost et al., 2005): N = ICC_t_ / (1 − ICC_t_) × [(1 − ICC_s_) / ICC_s_], where N = number of days needed, ICC_t_ = desired level of reliability, and ICC_s_ = reliability for single days.

Bland Altman plots, showing the difference between two subsequent weeks as a function of the mean of the two weeks (Bland and Altman, 1986), were applied to show the week-by-week measurement variability. Because the data were homoscedastic, 95% LoAs were calculated from the residual variance (i.e., within-subjects) error term obtained through a one-way random effect model using week-by-week data (LoA = √residual variance × √2 × 1.96) (Weir, 2005). Reliability for two weeks of measurement was estimated using variance partitioning obtained through a one-way random effect model (between subject variance / (between subject variance + (residual variance / 2))) (McGraw and Wong, 1996).

All analyses were performed using IBM SPSS v. 20 (IBM SPSS Statistics for Windows, Armonk, NY: IBM Corp., USA). A p-value < .05 indicated statistically significant findings.

## Results

### Subject characteristics

Of the 94 included children, 91 provided accelerometer data (49% boys; 28% 3-year-olds, 37% 4-year-olds, and 35% 5-year-olds). Weekly mean (SD) SED and PA across the two weeks were: Overall PA = 714 (157) cpm; SED = 335 (44) min/day, equal to 49.2 (5.5)% of the day; LPA = 281 (34) min/day, equal to 41.2 (4.3)% of the day; MVPA = 63 (20) min/day, equal to 9.2 (2.8)% of the day; LVPA = 344 (45) min/day, equal to 50.4 (5.5)% of the day. The guideline amount of PA was achieved for 55 and 100% of the children, according to the aim of achieving ≥ 60 min/day of MVPA and ≥ 180 min/day of LVPA, respectively.

### Reliability for the ≥ 6 to ≥ 10 hour criteria to define a valid day

The number of days that were available for analysis declined as a result of applying a more strict wear time criteria (n = 1070 [84%] for ≥ 6 h/day [19, 9, 16, 46 and 92 children had ≤ 3, 4, 5, 6 and 7 valid days, respectively]; n = 1011 [79%] for ≥ 8 h/day [20, 15, 27, 51 and 69 children had ≤ 3, 4, 5, 6 and 7 valid days, respectively]; n = 851 [67%] for ≥ 10 h/day [40, 25, 45, 42 and 30 children had ≤ 3, 4, 5, 6 and 7 valid days, respectively]). Table 1 shows the reliability for single days of measurement (ICC_s_) and the number of days (N) needed to achieve a reliability of 0.80, as estimated by the Spearman Brown prophecy formula. Reliability increased with a stricter wear time criteria: More than 7 days of measurement was needed to achieve the desired reliability for SED, LPA and LVPA (min/day) using the ≥ 6 and ≥ 8 hour/day criteria, whereas all variables could be reliably estimated using the ≥ 10 h/day criterion. The percentage values provided better reliability estimates than the absolute minutes per day, but differences were attenuated when a stricter wear time criterion was applied.

### Reliability for two consecutive weeks of measurement

We found slight improvements in week-by-week reliability when data was accumulated over longer days (≥ 6 to ≥ 10 h) and more days (≥ 3 to ≥ 5 d) (Table 2), however, the pattern was not fully consistent and the differences were minor. All criteria provided ICC estimates ≥ 0.75 for all outcome variables, except for SED reported as an absolute value (min/day), for which ICC varied from 0.61 to 0.81. The estimated ICC for applying two weeks of measurement were 0.87 for overall PA, 0.81 (min/day)/0.88 (%) for SED, 0.88 (min/day)/0.89 (%) for LPA, 0.93 (min/day)/0.92 (%) for MVPA, and 0.89 (min/day)/0.88 (%) for LVPA. The ICC and LoA for wear time varied from 0.53 to 0.73 and from 45 to 105 min/day across the criteria, respectively, with LoAs clearly decreasing as stricter criteria were applied.

**Fig. 1:**
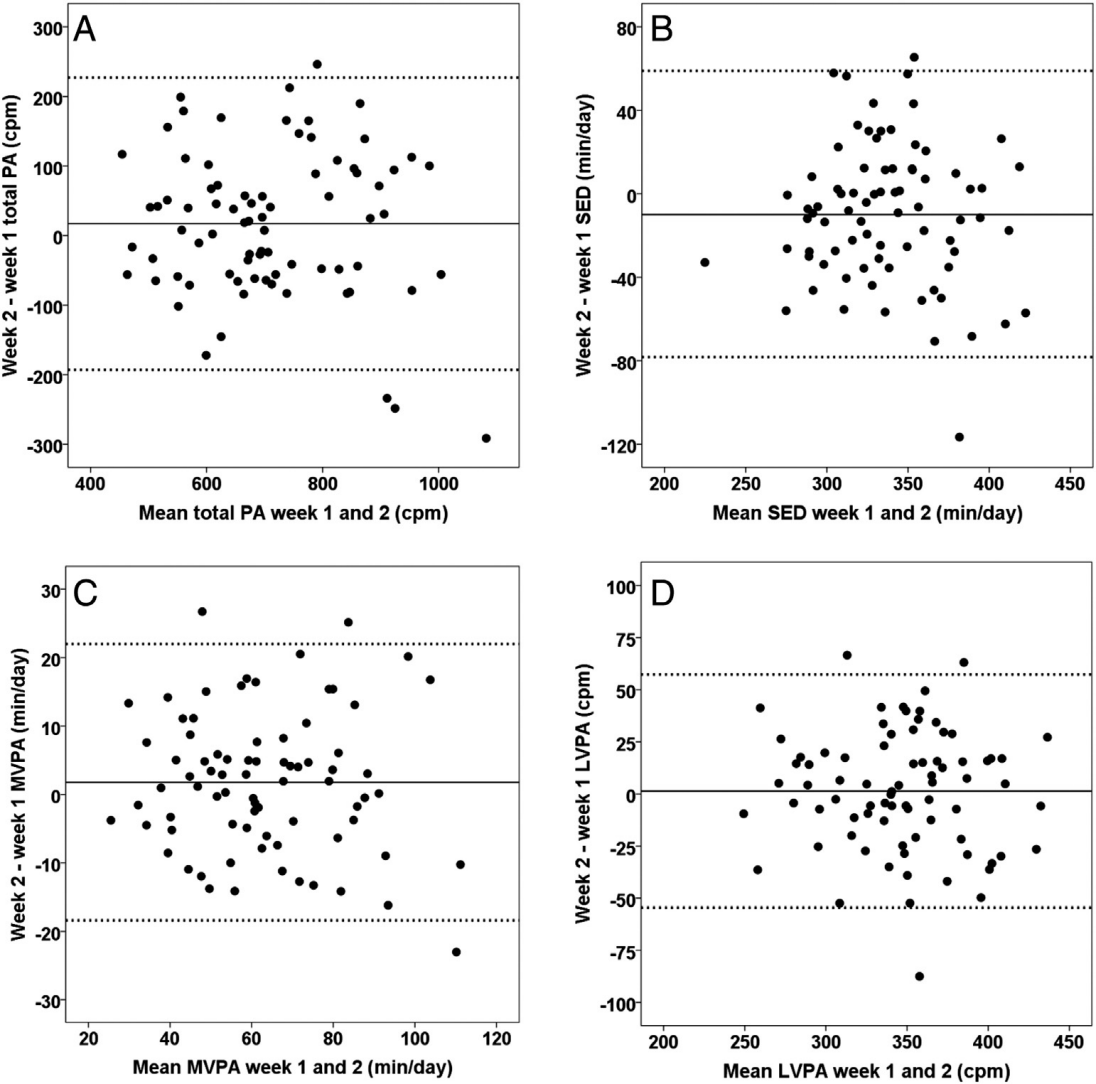
Bland Altman plots of agreement for different outcome variables over two consecutive weeks of measurement. Bland Altman plots (mean of two weeks of measurement on the x-axis versus the difference between them on the y-axis) for (A) overall physical activity (cpm) and (B) minutes per day spent sedentary (SED), (C) in moderate-to-vigorous physical activity (MVPA) and (D) in light-to-vigorous physical activity. Results are based on a ≥ 8 h & ≥ 3 days wear time criterion (n = 78). The full line is the bias between weeks, whereas the dotted lines are 95% limits of agreement. The study was conducted in Sogn og Fjordane, Norway, 2014.

Fig. 1 shows Bland Altman plots for overall PA, SED, MVPA, and LVPA using a ≥ 8 h & ≥ 3 days wear time criterion (week 1: wear time = mean (SD) 691 (42) min/day, wear days = 0, 4, 14, 25 and 35 children with 3, 4, 5, 6 and 7 valid days, respectively; week 2: wear time = 682 (45) min/day, wear days = 1, 7, 12, 24 and 34 children with 3, 4, 5, 6 and 7 valid days). Although all variables except SED (ICC = 0.69) reached an ICC equal to or above ~ 0.80, the absolute measures of reliability clearly showed that a substantial degree of individual variability must be expected across subsequent weeks. Across variables, the 95% LoAs were ± 1.0–1.6 times the sample SDs (MVPA = 1.0 SD; overall PA, LPA, LVPA = 1.2–1.3 SDs; SED = 1.6 SDs).

## Discussion

The present study is the first to investigate agreement of week-by-week measurements of SED and PA, as obtained by accelerometry in preschool children. Our findings indicate that the activity level of a given child should be expected to vary by up to ± 1.0 to 1.6 SD units from one week to another. Thus measurement error was substantial for all outcome variables.

By application of standard data reduction wear criteria (≥ 6–10 h/day and ≥ 3 and 5 days/week), we found reliability estimates ≥ 0.75 for all outcome variables, except for SED (min/day). Thus, in terms of ICC, our results were consistent with previous studies that have estimated reliability over one week of measurement in preschool- (Addy et al., 2014, Hinkley et al., 2012, Hislop et al., 2014, Penpraze et al., 2006) and older children (Basterfield et al., 2011, Chinapaw et al., 2014, Janz et al., 1995, Kang et al., 2009, Murray et al., 2004, Ojiambo et al., 2011, Rich et al., 2013, Treuth et al., 2003, Trost et al., 2000), which indicates generalizability to other study samples. Still, studies that have applied several measurement periods over the course of a year have yielded substantially lower reliability estimates in adults (Levin et al., 1999) and children (Mattocks et al., 2007, Wickel and Welk, 2010). Mattocks et al. (2007) determined overall PA, MVPA and SED over four 7-day periods over about one year using the Actigraph 7164 accelerometer in 11–12 year-old children. The ICC for one period of measurement varied from 0.45 to 0.59 across outcome variables. Wickel and Welk (2010) found an ICC of 0.46 for one out of three 7-day periods to assess steps for the Digiwalker pedometer in 80 children aged 9.8 (0.9) years. These findings question the validity of one week of measurement to determine people's habitual activity-level. As the present results were clearly superior to these findings, the agreement for habitual activity level over a year must be expected to be poorer than our findings indicate.

Our findings showed that reliability, in general, were lower for absolute measures (min/day) than for relative measures (%) of PA and SED. This is consistent with the great variability of wear time, as time in different intensity categories will co-vary with wear time (Herrmann et al., 2014). Thus, our findings show that outcomes should be corrected for wear time, either by using percentage values or by adjusting analyses for wear time, to maximize reliability. The pattern of increased reliability for SED along with a confined wear time (Table 2) is in line with the above argument.

As noise in exposure (x-) variables will lead to attenuation of regression coefficients (regression dilution bias), and noise in outcome (y-) variables will increase standard errors (Hutcheon et al., 2010), unreliable measures weaken researchers ability to make valid conclusions in epidemiology. Although an increased monitoring length might improve validity of study conclusions, the burden for subjects should be kept minimal to maximize response rate. Yet, we found minimal difference in wear time and valid days between week 1 and 2, and received a no complaint from our participants, which indicates that the 14-day protocol was well accepted. Also, the number of observations for analyses declined with increased wear time criteria, as shown previously (Colley et al., 2010). Thus, the choice of wear criteria is a trade-off between reliability and power, of which both are of crucial importance to avoid performing type II errors. In any case, monitoring volume needed is a matter of the research question posed, as population-estimates on a group level requires less reliability than individual-level estimates (Matthews et al., 2012).

### Strengths and limitations

We are the first to present absolute measures of agreement for PA and SED as obtained by accelerometry. As our findings, in terms of ICC, as well as overall PA level (Bornstein et al., 2011), were consistent with previous studies, we believe that the reported results are generalizable to preschool children in general. A limitation of the present study is that we only report reliability for the Evenson et al. (2008) cut points for SED, LPA, MVPA and LVPA. Which accelerometer cut points to apply in different populations is heavily debated, and the use of many different thresholds to determine the time spent in different intensities causes a certain degree of confusion across studies (Cain et al., 2013). The Evenson et al. (2008) cut points have been found to perform well in external validation studies in youth (5–15 years of age) (Trost et al., 2011) and preschool (4–6 years of age) (Janssen et al., 2013) samples. Janssen et al. (2013) also found the Pate et al. (2006) MVPA cut point (≥ 1680 cpm) developed in preschool children to perform well, however, applying this cut point to our data did not change any findings in terms of reliability.

Future studies should seek to verify the current findings and explore agreement for longer intermittent periods of accelerometer measurement across populations.

## Conclusion

We conclude that one out of two consecutive weeks of accelerometer monitoring in preschool children using standard wear criteria left modest agreement, despite the relative reliability being apparently good (ICC equal to or above ~ 0.80). Thus, considerable week-by-week variability was found. Because noise in any measurement will preclude researchers' ability to arrive at valid conclusions in epidemiology, researchers need to be aware of intra-individual variability in accelerometer-measurements and take appropriate actions according to the hypothesis under study. We encourage researchers to consider more than 7 days of accelerometer measurement in future studies involving preschool children to increase the reliability of the accelerometer measurements and increase the validity of the study conclusions.

## Conflict of interest statement

The authors declare that they have no competing interests.

## Figures and Tables

**Table 1:** Reliability for single days of measurement (ICCs) and number of days needed to achieve a reliability of 0.80 (N) for different criteria to determine a valid day of measurement.

	Wear time criteria for a valid day					
	≥ 6 h		≥ 8 h		≥ 10 h	
	ICC_s_	N	ICC_s_	N	ICC_s_	N
Overall PA (cpm)	0.37	7.0	0.38	6.6	0.38	6.4
SED (min/day)	0.30	9.5	0.32	8.6	0.37	6.7
SED (%)	0.38	6.5	0.39	6.1	0.42	5.6
LPA (min/day)	0.30	9.1	0.33	8.2	0.36	7.1
LPA (%)	0.42	5.5	0.43	5.3	0.46	4.8
MVPA (min/day)	0.45	4.8	0.48	4.3	0.51	3.9
MVPA (%)	0.48	4.3	0.49	4.2	0.51	3.9
LVPA (min/day)	0.31	8.9	0.34	7.6	0.37	6.9
LVPA (%)	0.38	6.5	0.39	6.1	0.42	5.6

CPM = counts per minute; SED = sedentary time; LPA = light physical activity; MVPA = moderate-to-vigorous physical activity; LVPA = light-to-vigorous physical activity; ICC_s_ = intraclass correlation for a single day of measurement; N = number of days needed to achieve a ICC = 0.80. The study was conducted in Sogn og Fjordane, Norway, 2014.

**Table 2:** The week-by-week reliability for different outcome variables for two consecutive weeks of measurement by different wear time and wear days criteria.

	≥ 6 h/day				≥ 8 h/day				≥ 10 h/day			
	≥ 3 days/week		≥ 5 days/week		≥ 3 days/week		≥ 5 days/week		≥ 3 days/week		≥ 5 days/week	
	ICC	LoA	ICC	LoA	ICC	LoA	ICC	LoA	ICC	LoA	ICC	LoA
n (%)	*83 (91)*		*70 (77)*		*78 (86)*		*67 (74)*		*72 (79)*		*47 (52)*	
CPM	0.78	209.0	0.75	209.7	0.78	209.5	0.76	200.3	0.70	242.3	0.76	219.5
SED (min/day)	0.61	78.5	0.61	75.1	0.69	68.6	0.72	61.3	0.68	69.5	0.81	50.0
SED (%)	0.76	7.6	0.78	7.1	0.78	7.2	0.80	6.6	0.75	7.8	0.78	6.9
LPA (min/day)	0.75	50.6	0.75	51.1	0.79	43.8	0.76	44.6	0.72	47.1	0.76	43.6
LPA (%)	0.79	5.4	0.83	4.9	0.81	5.3	0.83	4.8	0.77	5.8	0.83	5.0
MVPA (min/day)	0.84	22.1	0.85	21.0	0.87	20.2	0.86	20.0	0.85	22.2	0.85	22.8
MVPA (%)	0.83	3.1	0.83	3.1	0.86	2.9	0.86	2.8	0.83	3.3	0.85	3.1
LVPA (min/day)	0.76	64.9	0.76	64.3	0.80	55.9	0.77	56.7	0.75	58.9	0.74	57.8
LVPA (%)	0.76	7.6	0.78	7.0	0.78	7.2	0.80	6.6	0.75	7.7	0.78	6.9

CPM = counts per minute; SED = sedentary time; LPA = light physical activity; MVPA = moderate-to-vigorous physical activity; LVPA = light-to-vigorous physical activity; ICC = intraclass correlation; LoA = 95% limits of agreement. The study was conducted in Sogn og Fjordane, Norway, 2014.
